# Detailed Consideration of a Novel Meandered Dipole Array for Magnetic Resonance Imaging of the Head at 3 Tesla with Low Radiofrequency Power Deposition

**DOI:** 10.3390/s26123867

**Published:** 2026-06-17

**Authors:** Maryam Arianpouya, Benson Yang, Peter Truong, Simon J. Graham

**Affiliations:** 1 Physical Sciences Platform, Sunnybrook Research Institute, Toronto, ON M4N 3M5, Canada; benson.yang@sunnybrook.ca (B.Y.); peter.truong@sri.utoronto.ca (P.T.); 2 Department of Medical Biophysics, University of Toronto, Toronto, ON M5G 2C4, Canada

**Keywords:** MRI, meandered dipole antenna, parasitic elements, parallel radiofrequency transmission (pTx), specific absorption rate (SAR)

## Abstract

Electric dipole antennas can be designed in a variety of geometries and applied across a wide range of configurations. Appropriately designed dipole antennas can provide deep tissue penetration and low radiofrequency (RF) power deposition in magnetic resonance imaging (MRI), making them attractive for applications requiring safe and effective RF transmission in deep regions. On clinical 3 T MRI systems, however, conventional dipoles are too large in size for practical imaging of the head. Inspired by telecommunications designs, the present work adapts meandered dipoles (where the conductor is folded to shorten the antenna) with the resonance frequency controlled through trace geometry. Additionally, multi-channel configurations are considered to improve RF power transmission. A straight dipole was progressively transformed into meandered geometries and characterized using benchtop measurements and electromagnetic simulations. Analyses evaluated frequency response, near-field behavior, power-flow directionality, and distributions of local tissue heating and transmitted RF magnetic field in multi-channel arrays. A four-channel parallel-transmit (pTx) prototype was also used to show the feasibility of dipole-based head imaging at 3 T. The present work demonstrates a practical implementation of compact, low-heating dipole arrays for head MRI, with potential for extension to ultra-high-field or multinuclear imaging.

## 1. Introduction

**Figure 1:**
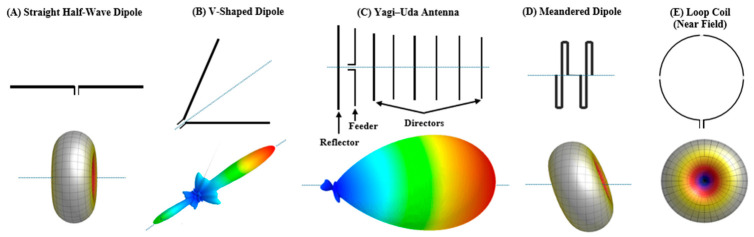
Far-field radiation patterns of four dipole-based antenna designs and the near-field pattern for a quarter-wavelength loop coil. (**A**) Straight half-wave dipole antenna with an omnidirectional pattern in the plane perpendicular to its axis. (**B**) V-shaped dipole antenna with directionality determined by the bend angle (adapted from [9]). (**C**) Yagi–Uda antenna with six director elements and one reflector (adapted from [10], licensed under GNU Free Documentation License v1.3.), showing enhanced forward gain and directivity. (**D**) Meandered dipole with reduced physical length, exhibiting a slight tilt of the main radiation lobe relative to the dipole axis when compared with the straight dipole. (**E**) A quarter-wavelength loop coil with maximum radiation strength near and parallel to the conductor. Note that colors of the field patterns are used to qualitatively illustrate the spatial differences in field propagation between the various antenna designs and the loop coil, rather than to enable quantitative comparisons.

The electric dipole antenna is widely used in radio and telecommunications. The typical implementation consists of a straight half-wave dipole with two identical linear elements, each with a physical length—the geometric end-to-end distance of the conductor—equal to one-quarter of the wavelength [1]. This structure is naturally resonant at odd multiples of the fundamental frequency ($f_{0}$, 3$f_{0}$, 5$f_{0}$, etc.) when the feed point is placed at the center, or at both even and odd multiples if fed asymmetrically (due to the disrupted current symmetry). The straight dipole produces an omnidirectional radiation pattern, with maximum radiation occurring in the plane perpendicular to the antenna axis, as illustrated in Figure 1A.

Various alternative antenna designs have been introduced to obtain better directivity of the emitted radiation. Notable examples include the V-dipole [1] and other modified dipole configurations [2], which alter the current distribution to achieve directional radiation, as well as Yagi–Uda antennas [3] and other parasitic-element-based designs [4,5,6,7,8], which enhance directivity and gain by incorporating additional passive elements such as directors and reflectors, strategically positioned relative to the driven dipole. The radiation patterns of a V-dipole antenna [9], and a Yagi–Uda antenna with six director elements and one reflector [10], are shown respectively in Figure 1B,C.

In applications requiring antenna size reduction, such as hand-held mobile devices, the dipole geometry is often modified into meandered (folded) structures [8,11,12,13,14]. Although some meandered geometries alter the radiation pattern [8,11,12,13,14], simply folding the elements results in a radiation pattern broadly similar to that of a straight dipole [13,14]. The primary effect of folding is a slight rotation of the main radiation lobe relative to the dipole axis, as illustrated in Figure 1D for a dipole with a single meander in each arm. Increasing the number of meanders progressively shifts the null axis, ultimately aligning it with the principal axis of the dipole.

Three distinct forms of “length” can be defined in such compact designs. The physical length (*L_p_*) refers to the end-to-end geometric distance that the antenna occupies in space (i.e., the overall one-dimensional footprint). The trace length (*L_c_*) represents the actual path of the conductor which would be obtained if the folded trace were straightened. In meandered dipoles, the trace length exceeds the physical length due to the folded geometry. The electrical length (*L_e_*) is the electromagnetic (EM) equivalent length that depends on the phase delay of current propagation, dielectric loading, and geometry. The *L_e_* value determines the resonant properties of the antenna and is a dimensionless parameter quantifying the length of the conductor measured in wavelengths of the fundamental frequency.

In comparison, the radiation pattern of a small loop coil (characterized by a circumference of much less than a wavelength, ~λ/4) is included in Figure 1E. Whereas antennae are used widely in communications applications and are designed for efficient far-field propagation, loop coils are key basic elements for transmission of excitatory radiofrequency (RF) fields and reception of RF signals from biological tissues in medical magnetic resonance imaging (MRI), commonly conducted at static magnetic fields of 1.5 T–3 T. Small loop coils operate predominantly in the near field, as required for MRI, with maximum RF magnetic field near the conductor and with good penetration into patient anatomy. Dipole-based antennas, by contrast, exhibit other flexible geometry-dependent radiation patterns (Figure 1) that, when adapted for MRI use, influence the spatial distribution of the transmitted RF field within the patient. This distinction is particularly relevant in present work, as introduced below.

Despite typical MRI practices, electric dipole designs have gained particular interest for emerging MRI applications at ultra-high field (UHF; ≥7 T). The corresponding Larmor frequency for UHF MRI is ≥298 MHz with the RF wavelength in biological tissue approaching human anatomical dimensions (e.g., ~12 cm at 7 T).

One of the major MRI concerns associated with conventional dipoles is their elevated specific absorption rate (SAR) of RF power deposition, and the associated risk of deleterious heating of tissues, especially near the feed point where electric fields are strongest [15,16]. Fractionated dipole arrays have been proposed to mitigate this issue, first by Raaijmakers [16,17] and further developed by several research groups [18,19,20,21]. These arrays segment the dipole into shorter elements connected via meandered or “S-shaped” conductors, which are often described in studies on MRI coil design as inductive elements [17,18]. This characterization is, however, an approximation. It does not fully reflect the distributed EM nature of the structure, which incorporates both inductive and capacitive effects, as well as current path redistribution along the conductors [22]. Nevertheless, meandering clearly reduces the physical length of the antenna, making it more practical for MRI applications, while appropriately modifying the current distribution and EM field behavior. By mechanisms that are not completely understood [23], local SAR hotspots are reduced and the RF transmission field (B_1_^+^) homogeneity in the region of interest (ROI) is improved without compromising transmit efficiency [17,18].

Several other studies have explored hybrid configurations that combine various dipole geometries: straight [24,25], fractionated [18,26], or otherwise modified [27], with loop coils [24,25], or monopole elements [28,29] to further optimize performance for specific UHF MRI targets such as the torso [18,24] and head [25,26,27,28]. More recently, dipole elements have also been combined with microstrip transmission line (MTL) resonators [30], which exhibit inherently low EM coupling with dipoles and therefore enable high-density array configurations with improved channel scalability and decoupling performance [30,31]. A typical MTL resonator consists of a conductive strip (usually copper) separated from a ground plane by a dielectric substrate, forming a transmission line. Additionally, an array of paired folded-end dipoles was introduced for whole-brain imaging at 9.4 T, whereby each driven dipole was paired with a nearby parasitic element [32]. Adjusting the overlap and reactive loading of the parasitic elements improved B_1_^+^ homogeneity and extended coverage—especially in hard-to-reach areas like the brainstem—compared with earlier single-row designs from the same group [33].

Despite their advantages at ultra-high field (UHF), dipole antennas have seen limited use to date for MRI at the lower fields of 1.5 T and 3 T. One major limitation is the large antenna size at these frequencies, with effective dipole lengths on the order of ~100 cm at 64 MHz (1.5 T) and ~50 cm at 128 MHz (3 T), depending on loading and geometry. These dimensions are impractical for imaging in clinical settings (and especially neuroimaging), where space constraints and patient comfort within the magnet bore are critical considerations.

The size-reduction techniques, such as meandering, that are commonly used in mobile communication antennas have also been adopted in early MRI dipole design work to address the physical length constraints at 3 T, as well as SAR concerns. For example, an 8-channel dipole array transceiver was proposed for prostate imaging at 3 T, with each element composed of serially connected S-shaped segments [34]. The array was tuned and matched using compact lumped-element networks at the feed point. In a follow-up study, the same group utilized this array with fixed-phase excitation on a standard 3 T MRI system, achieving T_1_- and T_2_-weighted abdominal images with relatively homogeneous signal intensity, though some localized signal voids were observed across multiple participants [35]. These results demonstrate the feasibility of dipole arrays at clinical static magnetic fields, extending their utility beyond UHF systems.

The present work considers in detail how to adapt and extend such dipole designs for brain MRI at 3 T. At the outset, dipole design for head imaging in this scenario must address two major considerations. First, tuning and matching dipole antennas for 3 T MRI at 128 MHz is technically demanding. In contrast to conventional MRI loop coils, which are typically tuned and matched using capacitor-based networks, dipole antennas often require a combination of inductors and capacitors, increasing tuning complexity and practical implementation effort [17]. Second, dipole antennas—regardless of operating resonance frequency—exhibit strong mutual coupling when used in multi-element arrays. Array configurations are essential in MRI applications to provide adequate volume of coverage. Additionally, each array element can be driven by a common input waveform or can be driven separately on MRI systems equipped with parallel RF transmission (pTx). The pTx capability provides more degrees of freedom for manipulating the transmitted magnetic and electric fields, for improved image uniformity and reduced SAR. Coupling effects become especially pronounced in arrays with many elements packed closely together, such as a 16-channel setup for UHF MRI designed to conform to the human head [26]. Some studies have intentionally taken advantage of this mutual coupling using the induced currents between neighboring dipoles to drive multiple elements from a single feed port [19]. However, strong mutual coupling remains a critical challenge in most practical array configurations [36], including those encountered in this project. Closely spaced dipoles tend to detune one another, causing resonance splitting (i.e., the appearance of two shifted resonance peaks instead of one). If this detuning is not corrected—typically through phase shimming (discussed in the Discussion) or dedicated decoupling measures—then it can significantly reduce B_1_^+^ penetration and homogeneity.

To address the first consideration, the present work adapts the concept of meandered dipoles from the telecommunications field, where resonance is controlled without lumped components by adjusting the electrical length through trace geometry and conductor path. This is implemented empirically by progressively transforming a straight dipole into increasingly meandered geometries and characterizing the resulting resonance behavior through both benchtop measurements and EM simulations.

To address the second consideration, intentional pairing is exploited for a certain distance between adjacent multi-channel dipole elements. After the meandered dipole is tuned to the target resonance frequency by adjusting *L_c_*, the driven element is paired with an identical neighboring element (while avoiding resonant coupling between different pairs). This approach is found empirically to increase transmit gain substantially.

Based on these approaches, and after initial proof-of-concept experiments investigating the effect of meandering of a straight dipole on resonance characteristics, EM simulations were conducted in six steps. The first three steps evaluated: (i) the |S_11_| frequency response of five unloaded dipole geometries with increasing meandering degrees of a straight dipole, (ii) the |S_11_| frequency response of a tightly meandered dipole loaded with a head model, and (iii) the near-field behavior of the same dipole in free space and within the head medium. Subsequent analyses focused exclusively on tightly meandered dipole pairs, including: (iv) their resonance behavior in free space and under head loading, (v) power-flow directionality, and (vi) SAR and |B_1_^+^| distributions in multi-channel tightly meandered dipole arrays, with comparison to loop arrays. Proper pairing at a defined distance was essential, as unpaired elements showed markedly reduced performance. This approach also demonstrates potential extension to UHF or multinuclear MRI applications, beyond the scope of the present work. Finally, a prototype of the optimized dipole configuration, implemented in a four-channel pTx system, provides the practical demonstration of dipole-based 3 T MRI using a head phantom.

## 2. Materials and Methods

### 2.1. Initial Experimental Observations

Straight half-wave dipoles were initially constructed from flexible tinned copper wires (solid, 1.29 mm diameter, equivalent to 16 American Wire Gauge), allowing easy reshaping into various zigzag and meandered configurations. The goal of the subsequent proof-of-concept experiments was to assess how the number of meanders, the folding angle, and the progressive transformation from straight to compact meandered geometries influenced the dipole frequency response. The specific aim was to verify physical dimensions of the meandered segments to achieve the fundamental resonance frequency of 123.25 MHz—the resonance frequency of the hydrogen nucleus for the 3 T MRI system used in the present work (MAGNETOM Prisma, Siemens Healthineers, Erlangen, Germany). Benchtop tests were conducted to explore resonance behavior using a network analyzer (E5061B, Keysight Technologies, Santa Rosa, CA, USA), without employing any lumped components.

### 2.2. Simulation Setup

**Figure 2:**
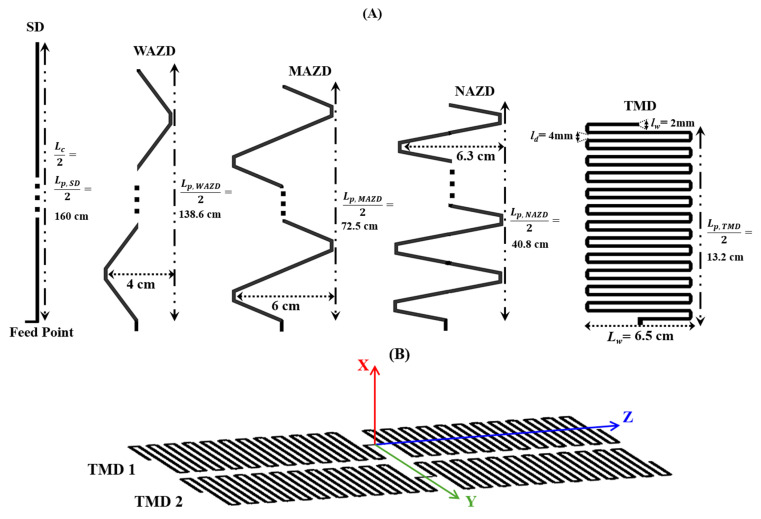
Simulation workflow for dipole geometry transformation. Five dipole designs were modeled with a fixed trace length (*L_c_*) of 160 cm for each arm and a copper trace width (*l_w_*) of 2 mm. (**A**) Each of the 5 designs are illustrated showing single-arm dimensions and central feed point. Starting from the straight dipole (SD), progressive angular folding generated the wide-angle zigzag dipole (WAZD), moderate-angle zigzag dipole (MAZD), narrow-angle zigzag dipole (NAZD), and finally the tightly meandered dipole (TMD) featuring twelve meanders on each arm. Folding was applied symmetrically, reducing physical length (*L_p_*) while maintaining constant *L_c_*. (**B**) Simulation model of the TMD 1 paired with an identical element (TMD 2) in free space, in the same YZ plane. The origin of the coordinate system is located at the feed point of TMD 1.

Next, EM simulations were performed using Sim4Life (Zurich MedTech, Zurich, Switzerland) to evaluate five distinct dipole geometric configurations (Figure 2A).

**Figure 3:**
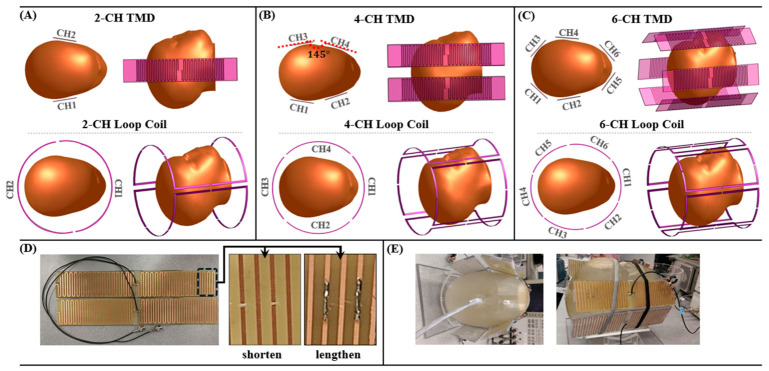
(**A**) Simulation setup for near-field evaluation. Two TMDs were placed symmetrically in an opposing-element configuration, with a tissue-mimicking head model at the MRI system isocenter as shown in top and side views. Each TMD arm contained twelve meanders. For comparison, a two-channel saddle loop-coil array was also positioned around the head model. (**B**,**C**) Analogous 4-channel (two-pair) and 6-channel (three-pair) setups for comparing TMD and loop-coil geometries, respectively. In (**B**), the red dotted lines indicate TMD pair elements angled slightly to conform with the head surface. (**D**) Fabricated TMD pair with tuning and matching demonstration: effective trace length (*L_c_*) is adjusted by cutting copper to shorten the dipole (**left**) or re-soldering meanders to lengthen it (**right**). (**E**) Experimental setup: four-channel TMD transceiver array used in phantom measurements, matching the simulation configuration shown in (**B**).

These included a straight dipole (SD), defined by a fixed *L_p_*/2 of 160 cm per arm (with *L_c_ = L_p_* = 320 cm), as determined from benchtop measurements, and a copper trace width (*l_w_*) of 2 mm. Angular bends were then progressively introduced to create a wide-angle zigzag dipole with twelve bends per arm (WAZD, *L_p_*/2 = 138.6 cm). By decreasing the bend angles successively, the design transitioned into a moderate-angle zigzag dipole (MAZD, *L_p_*/2 = 72.5 cm), a narrow-angle zigzag dipole (NAZD, *L_p_*/2 = 40.8 cm), and ultimately a tightly meandered dipole (TMD, *L_p_*/2 = 13.2 cm). Across all configurations, the total *L_c_* value was held constant, whereas *L_p_* reduced due to the increasingly compact, folded geometry, applied symmetrically to both dipole arms. The feed point was located at the geometric center of each dipole to ensure symmetric power transmission and prevent resonance splitting. The *l_w_* value of 2 mm was selected based on a maximum simulated current of approximately 6 A per arm for the most compact configuration (TMD), as estimated using Altium Designer (Altium LLC, San Diego, CA, USA), and was kept constant across geometries for consistency. Compacting the TMD to span approximately 30 cm (suitable for head coverage), with a conductor thickness of 2 mm and 4 mm (*l_d_*) spacing between adjacent traces, resulted in twelve meanders per arm and a projected width $L_{w}$ of approximately 6.5 cm. Simulations were performed in six steps:

**(i)**  **Frequency Response of Five Unloaded Dipole Geometries:** The |S_11_| frequency response of each of the five dipole geometries shown in Figure 2A was evaluated in free space.
**(ii)**  **Frequency Response of a TMD Loaded with a Head Model:** Among the five geometries investigated above, the most compact design (the TMD) was selected for subsequent simulations and experiments due to its reduced physical length and improved compatibility with human head anatomy (<30 cm across the two arms). To assess the feasibility of imaging with a single TMD dipole at 3 T, the TMD was loaded with a head model positioned at separations of 5, 10, and 20 mm from the dipole (configured similarly to that shown in Figure 3A but without the opposing TMD channel). The |S_11_| frequency responses corresponding to the three separations were recorded. To match simulation and experimental conditions, both the homogeneous head model and corresponding head phantom had the same dimensions and the same EM properties (conductivity = 0.46 S/m; relative permittivity = 80) representative of brain tissue [37].
**(iii)**  **Near-Field Evaluation of a TMD in Free Space and Inside Head Medium**. Using the step (ii) configuration with the TMD positioned 5 mm from the head model, the spatial characteristics of the $\left|{B_{1}}^{+}\right|$ field were evaluated near the dipole and within the head medium. The $\left|{B_{1}}^{+}\right|$ field distribution was normalized by the square root of the accepted input power ($P_{acc}$), the net power delivered to the coil port, to determine transmit efficiency (i.e.,$\left|{B_{1}}^{+}\right|/\sqrt{P_{acc}}$). The analysis was performed in three orthogonal planes, as defined by the coordinate system shown in Figure 2B: (1) the Y–Z plane, parallel to the dipole plane; (2) the X–Z plane, perpendicular to the dipole plane; and (3) the X–Y plane, perpendicular to both the dipole plane and the dipole axis.
**(iv)**  **Inter-Element Interaction of a Coplanar TMD Pair**. To investigate inter-element coupling effects on resonance behavior, a driven TMD (TMD 1) was placed adjacent to an identical TMD element (TMD 2) in the same plane to form a coplanar TMD pair (Figure 2B), and the |S_11_| frequency response of the pair was evaluated in free space. TMD 2 was configured under two excitation conditions:
  (a) as a parasitic element, terminated with a resistive load (50–90 Ω) with the optimal resistance determined by the input impedance of the driven dipole. An ideal center-fed half-wave dipole exhibits an input impedance of approximately 73 Ω in free space [1]; in practice, baluns are commonly used to match the dipole to a 50 Ω transmit system [1].
  (b) as an actively driven element, with a relative phase corresponding to effective input impedance of its pair. This phase was determined by sweeping the phase angle and selecting the value that maximized inter-element decoupling between elements in a pair.
  Subsequently, the coplanar TMD pairs were loaded with the head model, and their resonance behavior was evaluated for both excitation conditions.
**(v)**  **Directionality**. Next, the two configurations described in step (iv) were evaluated with and without the head model. The directionality of RF power was quantified using the Poynting vector, S = E × H (W/m^2^), to assess whether EM power was preferentially directed toward the head medium or radiated outward, with E (V/m) and H (A/m) denoting the electric and magnetic field strength vectors, respectively. Two excitation conditions were evaluated:
  (a) a driven TMD–parasitic pair, in which one element was actively driven and the adjacent element acted as a parasitic element (i.e., a 1Tx/2Rx configuration, where Tx denotes transmission and Rx denotes reception); and
  (b) a driven TMD pair, in which both elements were actively driven to form a two-channel transceiver system (i.e., a 2Tx/2Rx configuration).
**(vi)**  **SAR and** $\left|{B_{1}}^{+}\right|$ **Assessment of TMD vs. Loop Arrays**. After characterizing the resonance behavior, near-field properties, and directionality of the TMD configurations, $\left|{B_{1}}^{+}\right|$ efficiency, SAR efficiency and local ${SAR}_{10g}$ were evaluated in array configurations relevant for MRI applications. Transmit efficiency was calculated as described in Step (iii). SAR efficiency was defined as $\left|{B_{1}}^{+}\right|/\sqrt{{SAR}_{10gmax}}$, where ${SAR}_{10gmax}$ is the peak 10 g-averaged local SAR across the head volume. Two, four- and six-channel fully active TMD arrays (opposing elements, and two- and three-pair configurations; all channels driven, no parasitic elements) were applied to the head model, as shown in Figure 3A–C. The spacing between adjacent elements was fixed at the optimal separation determined in Step (iv). As commonly undertaken in pTx applications, an “RF shimming” procedure was performed at 123.25 MHz by optimizing the relative transmit phases between dipole elements to maximize $\left|{B_{1}}^{+}\right|$ efficiency across the entire head. All ${SAR}_{10g}$ maps were computed from the same optimized $\left|{B_{1}}^{+}\right|$ distributions, normalized to 1 W of $P_{acc}$, and therefore corresponded to identical excitation levels, enabling a fair comparison between the two-, four- and six-channel configurations. For comparison, the same simulation and optimization strategy was applied to saddle loop coil arrays arranged in two-, four- and six-channel configurations (Figure 3A–C). The saddle loop coil arrays had a height of 250 mm, and their width was determined by the number of elements such that the array spanned the full circumference. This setup was intended to compare the potential differences in transmit efficiency, field penetration, homogeneity and SAR performance between the TMD and loop geometries.
  In addition to the primary simulations described above, two supplementary analyses were conducted to further validate the results. First, to provide a reference comparison with a conventional volume coil, the same |B_1_^+^| efficiency, SAR efficiency, and ${SAR}_{10g}$ maps were computed for a birdcage coil driven in quadrature (CP) mode and loaded with the uniform head model, using an identical normalization procedure. Second, to assess the robustness of the four-channel TMD results with respect to tissue heterogeneity, the simulation was repeated using the MIDA (Multimodal Imaging-Based Detailed Anatomical) [38] head model in place of the uniform phantom.

All Sim4Life simulations were performed using a finite-difference time-domain (FDTD) solver with a uniaxial perfectly matched layer (UPML) applied at the boundaries of the computational domain [39]. The UPML is a standard absorbing boundary condition that mimics open space by absorbing outgoing EM waves with minimal reflection, which could otherwise distort current distributions and input impedance (S_11_).

### 2.3. Experimental Setup

Following simulations with two-, four-, and six-channel fully active TMD arrays, the four-channel configuration (i.e., two TMD pairs) was selected based on the simulation results and for its simpler implementation in proof-of-concept experiments. Four identical TMD elements were fabricated in-house by etching a single-sided copper-clad FR-4/G10 substrate (thickness = 0.81 mm; copper weight ≈ 170 g/m^2^, corresponding to ~17 µm thickness) using a ferric chloride etching solution. To maintain structural stability, all elements were affixed to a rectangular polyacrylic substrate. The feed point for each element was placed at the center, analogous to the corresponding simulation setup. All dipoles were fed via coaxial cables, with balun circuits incorporated to decouple the antennas from the feed lines—minimizing the influence of cable length and suppressing unwanted surface common-mode currents. Figure 3D shows two fabricated TMDs, along with the tuning and matching procedure. Tuning and matching were achieved by adjusting the effective *L_c_* length: shortening the trace by cutting the copper or lengthening it by re-soldering previously cut meanders. Each active TMD element was tuned to 123.25 MHz and matched to a 50 Ω source by introducing a symmetric cut in both dipole arms to adjust the number of meanders. In agreement with the simulations, twelve meanders per arm were required to achieve the target resonance frequency.

In-vitro 3 T MRI was then performed following the simulation setup described in Section 2.2 (vi), using a four-channel TMD array and a head phantom matched to the simulation model in geometry, element positioning, and electrical properties. The experimental setup is shown in Figure 3E. Adjacent TMD elements were intentionally paired, as described in Section 2.2 (iv) and (v), with an inter-element spacing of ~3 cm. In preliminary experiments, this spacing was found to be sufficient to enhance transmit gain while preventing resonance splitting and inter-element detuning.

During MRI, all four TMD elements were operated as driven antennas with each acting as a separate transceiver channel. The array can also function in a two-transmit/four-receive mode, in which only one element of each TMD pair is active during transmission. However, this configuration is beyond the scope of the present EM simulations.

Imaging was conducted using a four-channel custom-built parallel-transmit (pTx) platform integrated into a 3 T MRI system. A block diagram of the setup is provided in [40]. Each channel was powered by an independent 1 kW RF amplifier.

Initially, a magnitude-shimming calibration procedure was performed on a head phantom using “localizer” imaging (i.e., at low spatial resolution, prior to prescribing MRI with the intended spatial resolution). This calibration measured the actual transmit magnitude response of the pTx system as delivered through the MRI system. During the procedure, the transmit magnitudes were incrementally stepped while the output was monitored on an oscilloscope to (1) determine the actual |B_1_^+^**|** field and resulting flip angles; and (2) ensure that RF power levels did not exceed the limits of the RF power amplifiers.

Subsequent MRI of the phantom was undertaken using a gradient-recalled echo (GRE) sequence (repetition time TR = 2000 ms, echo time TE = 2.45 ms, slice thickness = 3 mm, acquisition matrix = 320 × 320, FOV = 220 mm, in-plane resolution = 0.68 mm × 0.68 mm, imaging acquisition time = 2:15 min) with two flip angles of 30° and 60° following the double angle method (DAM) for quantitative B_1_^+^ mapping [41]. Additionally, temperature changes were monitored using three fiber-optic temperature probes (OTG-MPK5, Opsens Solutions, Québec City, QC, Canada) with ±0.3 °C total accuracy (including both signal conditioner and sensor errors) placed at: (1) the feed point of active element CH1, (2) the feed point of active element CH2, and (3) the isocenter of the head phantom.

## 3. Results

### 3.1. Initial Experimental Observations

Using the initial stepwise experimental approach described in Section 2, the EM effects of folding a straight dipole into a compact meandered geometry were examined. By decreasing the *L_p_* value while maintaining a constant *L_c_* value, several key changes were observed: (1) the resonance peaks shifted to higher frequencies; (2) the spacing between successive resonant peaks did not remain constant within the observable frequency range of the network analyzer, in contrast to a straight dipole antenna displaying odd-harmonic resonances; (3) increasing *L_c_*, regardless of *L_p_*, shifted the resonance peaks to lower frequencies; (4) introducing any asymmetry into the arm lengths of the dipole split the resonance peaks; and (5) |S_11_| values decreased significantly with progressive folding. However, placing a duplicate of the dipole under test coplanar with the driven dipole substantially restored |S_11_| values without altering the resonance frequencies. Coplanar placement refers to positioning the duplicate dipole at the same height with a lateral separation, as shown in Figure 2B, and (6) loading the dipoles with phantoms shifted all peaks to lower frequencies, with the shift increasing as the phantom was moved closer to the dipole setup.

### 3.2. Simulation Results

#### 3.2.1. Frequency Response of Five Unloaded Dipole Geometries

**Figure 4:**
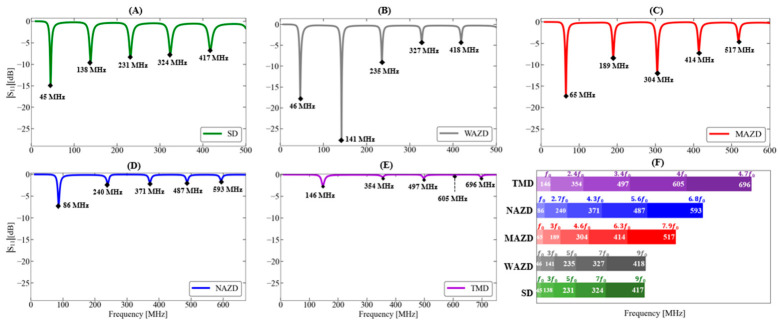
(**A**–**E**) Simulated reflection coefficient (|S_11_|) plots for the five unloaded dipole configurations shown in Figure 2A: SD (green), WAZD (gray), MAZD (red), NAZD (blue), and TMD (violet). (**F**) Bar plot summarizing the resonance behavior, showing the fundamental frequency ($f_{0}$) and associated higher harmonics across the dipole geometries. SD = simple dipole; WAZD = wide-angle zigzag dipole, MAZD = moderate-angle zigzag dipole, NAZD = narrow-angle zigzag dipole, and TMD = tightly meandered dipole.

The reflection coefficients for all five unloaded dipole geometries (Figure 2A) are plotted in Figure 4A–E, covering the fundamental frequency $f_{0}$ and the first four higher-order harmonics. Theoretically, the location of $f_{0}$ for the basic SD geometry can be estimated using the standard half-wavelength approximation [1]:

(1) $$f_{0}=\frac{c}{2L_{p}},$$

where *c* is the speed of light. In practice, *L_p_* of a dipole antenna is slightly shorter than the ideal half-wavelength due to wire thickness and end effects. Using the empirical relation $f_{0}=1.43\times 10^{4}/L_{p}$ (with *L_p_* in cm) [42], $f_{0}$ for the SD geometry was estimated to be 45 MHz. The higher-order odd harmonics are then located at 135 MHz, 225 MHz, 315 MHz, and 405 MHz, corresponding to 3$f_{0}$, 5$f_{0}$, 7$f_{0}$, and 9$f_{0}$, respectively. These values align well with the simulated reflection profile (observed at 45 MHz, 138 MHz, 231 MHz, 324 MHz, and 417 MHz), and the frequencies measured during benchtop measurements. For the remaining four geometries, all harmonic resonances shifted to higher frequencies. For example, $f_{0}$ increased to 46 MHz, 65 MHz, 86 MHz, and 146 MHz for the WAZD, MAZD, NAZD, and TMD, respectively. The corresponding fundamental and higher-order resonances for each geometry are also annotated for the simulated reflection coefficient data plotted in Figure 4. Additionally, Figure 4F shows bar chart data to clarify further the differences in fundamental frequency and higher harmonics among the antenna geometries. As the dipoles progressed from simple, to progressively more zigzag, to tightly meandered, the increase in the fundamental frequency was accompanied by progressive departure from odd-integer harmonics. Over this progression, the higher harmonics shifted downward in frequency, occurring at non-integer multiples of the fundamental and becoming relatively closer spaced. For example, the first five resonances of the TMD geometry occurred at $f_{0}$ *=* 146 MHz, 2.4$f_{0}$, 3.4$f_{0}$, 4$f_{0}$, and 4.7$f_{0}$.

Table 1 lists the Q-factor values that were calculated from the simulated reflection coefficient data for all five dipole geometries. The Q factor was calculated as the center resonance frequency divided by the −3 dB bandwidth (${BW}_{-3dB}$) of the reflection coefficient. Among the investigated geometries, the WAZD configuration exhibited generally higher Q-factor values at all observable resonances (except compared with the other geometries for which Q could be determined. For the NAZD geometry, a Q factor could only be calculated for the fundamental resonance because the −3 dB bandwidth was not measurable for the higher harmonics. No Q-factor estimation was possible for the TMD geometry for the same reason.

These collective results agreed with the key trends identified during benchtop testing, further validating the observed behavior.

#### 3.2.2. Frequency Response of a TMD Loaded with the Head Model

**Figure 5:**
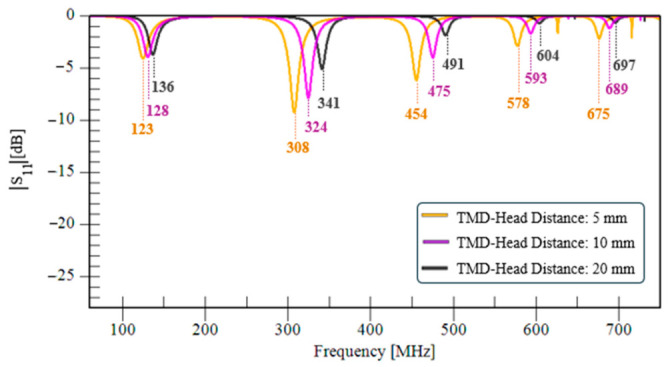
Simulated |S_11_| frequency response of a single TMD loaded with the head model at three separations: 5 mm, 10 mm, and 20 mm.

A TMD was loaded with the head model at separations of 5, 10, and 20 mm, as described in the second step of the simulation workflow. The corresponding simulated |S_11_| resonance peak locations were extracted and are presented in Figure 5. Loading caused two main effects: First, the resonance frequencies decreased, although the magnitude of the shift was not uniform across harmonic modes. For the fundamental mode, the resonance frequency decreased from 146 MHz in free space to approximately 123, 128, and 136 MHz for separations of 5, 10, and 20 mm, respectively. Second, a moderate increase in the reflection coefficient magnitude was observed, with |S_11_| for the fundamental mode increasing from approximately 2.5 dB for a single TMD in free space to about 4 dB at 5 mm separation under head loading. This behavior is illustrated by comparing Figure 4E (unloaded TMD) with Figure 5 (loaded TMD). Q factors for the loaded single TMD could not be reliably estimated due to small variations in |S_11_| across the three different separations.

#### 3.2.3. Near-Field Evaluation of a TMD in Free Space and Inside Head Medium

**Figure 6:**
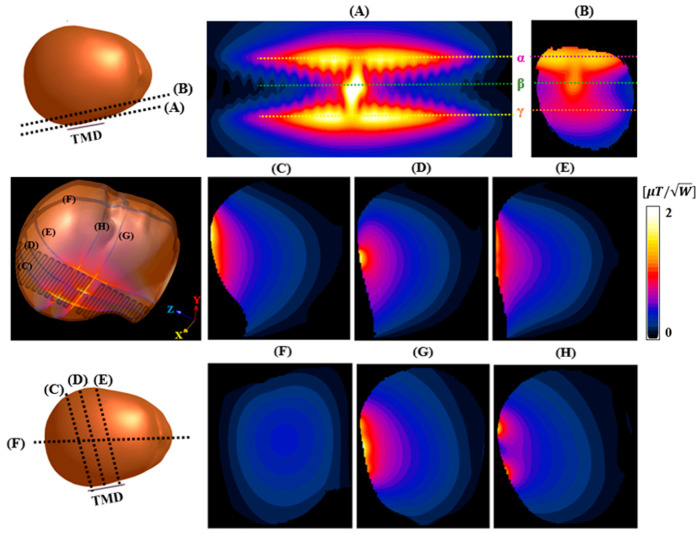
Simulated |B_1_^+^| efficiency maps obtained using the single-channel, head-loaded TMD element across eight sections, as illustrated schematically on the left column. (**A**) |B_1_^+^| efficiency map in the Y-Z plane adjacent to the TMD element, showing three distinct regions labeled α (top), β (midline), and γ (bottom). (**B**) |B_1_^+^| efficiency map in the same Y-Z plane as (**A**), but at 2 cm penetration depth into the head model. (**C**–**E**) X–Z |B_1_^+^| field maps perpendicular to the α (posterior; top of dipole), β, and γ (anterior; bottom of dipole) regions, respectively. (**F**) |B_1_^+^| efficiency maps at the head-model isocenter (sagittal section, 10 cm penetration). (**G**,**H**) Axial |B_1_^+^| efficiency maps at the TMD isocenter and 3 cm from the isocenter in the superior direction (toward the top of the head).

Using the simulation setup described in Section 2.2 (iii), the |B_1_^+^| field of a single-channel head-loaded TMD element was evaluated. Figure 6 (left column) provides a schematic overview of all sections used for |B_1_^+^| field extraction. Figure 6A shows the |B_1_^+^| field in the Y-Z plane adjacent to the dipole, with three distinct regions labeled α (top), β (midline), and γ (bottom). The |B_1_^+^| field near the dipole tails is nearly zero, consistent with the current distribution approaching zero at the ends of the dipole. Figure 6B shows the corresponding field distribution at a parallel section with a penetration depth of 2 cm into the head model.

In free space, a TMD produces a linearly polarized B_1_-field which is decomposed into equal contributions of right (B_1_^+^) and left (B_1_^−^) circular polarization, producing a symmetric field pattern (Figure 6A). This symmetry is observed in the α and γ components, which show similar |B_1_^+^| spatial distributions; a more detailed discussion is provided later in Section 4.2.1. At greater penetration depths (Figure 6B), the |B_1_^+^| field along the γ component attenuates more rapidly compared to the decrease observed in the α component of the |B_1_^−^| field. In a conductive medium, the applied B_1_-field induces a current which generates an out-of-phase magnetic field which adds location-dependent contributions to the overall B_1_-field. This results in constructive interference for one circular component and destructive interference for the other, yielding the observed asymmetric |B_1_^+^| distributions. Similar behavior has been reported for loop coils [43,44].

Because the |B_1_^+^| field distribution varies across planes relative to the TMD isocenter, |B_1_^+^| maps are shown for six additional sections. Figure 6C–E show the |B_1_^+^| field maps for sections perpendicular to lines α, β, and γ (defined in the Y–Z plane) and extending into the head model along the X–Z plane, respectively. These sections illustrate how the |B_1_^+^| field penetrates into the head at each of the three positions along the TMD. Figure 6C shows a larger |B_1_^+^| field near the head surface compared with Figure 6E, reflecting differences in field penetration along the α and γ lines. Figure 6D shows a large |B_1_^+^| field at the center, as this section is perpendicular to the β line and corresponds to the feed point. As the distance into the medium increases, the |B_1_^+^| field decreases substantially relative to the value at the surface. This behavior is illustrated in Figure 6F, which shows the |B_1_^+^| field in the sagittal section at the isocenter of the head model, located 10 cm from the head surface, where the average |B_1_^+^| field is approximately 0.5 µT/$\sqrt{W}$.

Figure 6G,H show the extracted axial |B_1_^+^| field distributions at the TMD isocenter and 3 cm in the superior direction (toward the top of the head). Even though the two arms of the dipole are separated by at least one meander (~1 cm), the |B_1_^+^| fields along the α and γ lines add constructively, effectively connecting the two arms. This behavior appears as three larger-intensity |B_1_^+^| regions in Figure 6G and two larger-intensity regions in Figure 6H. The region near the anterior portion of the head (toward the nose) has lower intensity compared with the region near the posterior portion, toward the back of the head.

Additionally, the TMD width (*L_w_*) was reduced to investigate |B_1_^+^| homogeneity inside the medium. Reducing *L_w_* from 6.5 cm to 1 cm while increasing the number of meanders to 35 per arm (at fixed physical length) caused the α and γ fields in the |B_1_^+^| distribution to merge, partially eliminating the midline β region. This modification shifted the fundamental resonance to 298 MHz and produced a more uniform |B_1_^+^| distribution within the medium. Further compaction to reach 128 MHz would require substantially thinner (*l_w_*) traces, which cannot safely carry MRI currents and may increase dipole heating, limiting practical implementation.

#### 3.2.4. Inter-Element Interaction of a Coplanar TMD Pair

Using the dipole orientations described in Section 2.2 (iv), two identical TMDs (TMD 1 and TMD 2) were next positioned adjacent to each other in free space and then loaded with the head model. Two excitation configurations were investigated: a coplanar TMD–parasitic pair and a coplanar driven TMD pair.

**Figure 7:**
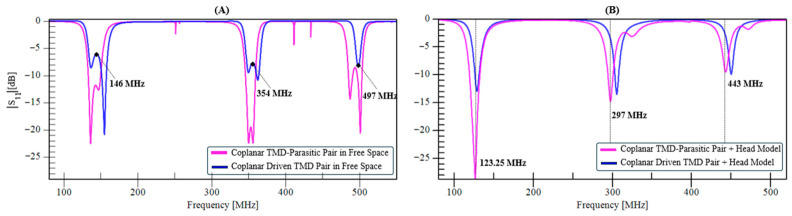
(**A**) Simulated |S_11_| frequency responses of a coplanar TMD–parasitic pair (pink) and a coplanar driven TMD pair (blue) in free space. (**B**) |S_11_| Frequency responses of the same configurations loaded with the head model.

For the free space scenario, the addition of TMD 2 modified the resonance behavior across all observed peaks and led to a substantial increase in |S_11_| for both excitation configurations. The magnitude of these effects depended on the inter-element separation and dipole length, as well as on the termination resistance in the TMD–parasitic case and the relative phase of excitation in the TMD 1 case. At an inter-element spacing of 3 cm, the simulated |S_11_| frequency responses of the first three resonance peaks are shown in Figure 7 for the TMD–parasitic pair terminated with a 70 Ω load and for the driven TMD pair excited with a 20° phase difference. At $f_{0}$ ~146 MHz, |S_11_| increased from approximately 2.5 dB for a single TMD (Figure 4E) to ~20 dB for both paired dipole configurations in free space (Figure 7A), with very little peak splitting (i.e., there was substantial peak overlap). Additionally, the mutual inter-element coupling between co-planar elements was approximately −3 dB in both paired dipole configurations.

When the coplanar TMD pairs were loaded with the head model, resonance behavior consistent with Figure 5 was observed, with loading shifting the fundamental resonance from 146 MHz in free space to approximately 123.25 MHz at a 5 mm separation, matching the operating frequency of the 3 T MRI system in this study (Figure 7B). Notably, resonance splitting was negligible for both TMD–parasitic pair and coplanar driven TMD pair configurations, with substantial |S_11_| values and Q factors (Table 2). The mutual inter-element coupling for the coplanar driven TMD configuration in the loaded case remained similar to the unloaded case (approximately −4.4 dB).

Additionally, it was observed that the UPML did not fully suppress boundary reflections for the 6.5 cm-wide TMD geometry used in this study. As a result, spurious peaks appeared in the |S_11_| frequency response (notably at 255, 413, and 463 MHz in Figure 7A) corresponding to minor false resonances that were not associated with true radiating modes.

#### 3.2.5. Directionality

**Figure 8:**
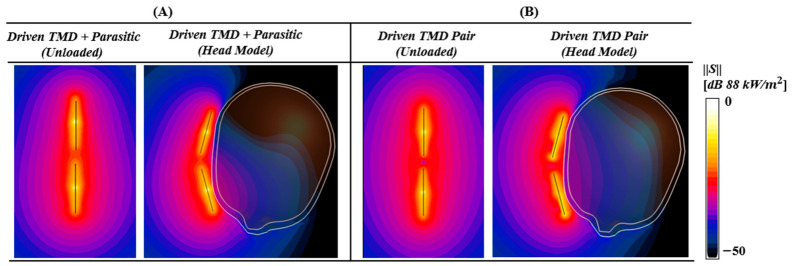
Poynting vector magnitude (‖S‖) maps for (**A**) a driven TMD–parasitic pair and (**B**) a driven TMD pair of identical geometry. Unloaded and head model-loaded conditions are shown for each TMD pair configuration.

As described in Section 2.2 (v), Poynting vectors were next simulated and the resulting magnitude maps (‖S‖) are shown in Figure 8 for (A) a driven TMD–parasitic pair and (B) a driven TMD pair. For each configuration, two conditions are presented in Figure 8A,B: unloaded and head-loaded. In the loaded configurations, the dipoles were slightly angled to conform to the head geometry (see Figure 3B). This placement, compared with coplanar, had no significant effect on field penetration when the elements were positioned laterally. In the unloaded condition, the driven TMD–parasitic pair showed mild spatial asymmetry in the ‖S‖ maps, with the parasitic element radiating less than the driven element. In the loaded condition, ‖S‖ maps showed pronounced directionality away from the head model and much less penetration within the head model. The driven TMD pair showed slightly more penetration than the driven TMD–parasitic pair. The driven TMD pair was therefore selected for all subsequent array simulations.

#### 3.2.6. SAR and |B_1_^+^| Assessment of TMD vs. Loop Arrays

As described in Section 2.2 (vi), the |B_1_^+^| efficiency, SAR efficiency, and local $SAR_{10g}$ were evaluated for two-, four- and six-channel head-loaded TMD arrays (opposing elements, and two- and three-pair configurations, without parasitics). The spacing between adjacent elements in each pair was fixed at 3 cm, corresponding to a mutual inter-element coupling of approximately −3 dB.

**Figure 9:**
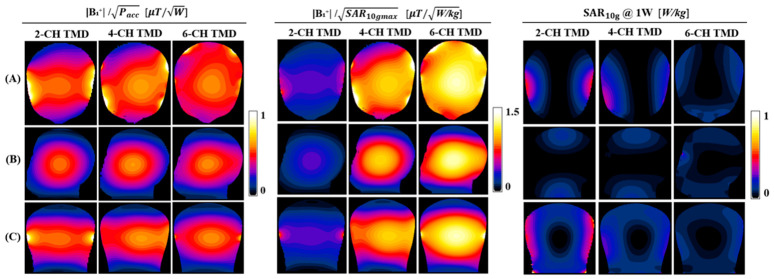
Simulated RF-optimized |B_1_^+^| efficiency (**left**), SAR efficiency (**middle**), and $SAR_{10g}\;$maps at 1W $P_{acc}$ (**right**) in the (**A**) axial, (**B**) sagittal, and (**C**) coronal sections at the isocenter for the TMD simulation setup shown in Figure 3A–C. Results are shown for two-channel (2-CH), four-channel (4-CH) and six-channel (6-CH) TMD arrays. $SAR_{10g}$ maps are displayed on a scale capped at 1 W/kg for visibility. Note that the FDA-defined global head SAR limit is 3.2 W/kg. $P_{acc}$ denotes the accepted input power.

The resulting optimal phase settings for the TMDs were [**2-CH: φ_1_ = 0°, φ_2_ =180°**], **[4-CH: φ_1_ = 0°, φ_2_ = 0°, φ_3_ = 180°, φ_4_ = 250°]** and **[6-CH: φ_1_ = 0°, φ_2_ = 0°, φ_3_ = 120°, φ_4_ = 120°, φ_5_ = 240°, φ_6_ = 240°]**. The optimal phase settings for the 2-CH and 6-CH configurations corresponded to CP mode excitation. The resulting |B_1_^+^| efficiency, SAR efficiency, and local $SAR_{10g}$ maps are shown in Figure 9 at three locations: (1) the axial section at isocenter of the head model (Figure 9A), (2) the sagittal section at isocenter (Figure 9B), and (3) the coronal section at isocenter (Figure 9C). Additionally, the corresponding maps for the 4-CH configuration in CP mode **(φ_1_ = 0°, φ_2_ = 0°, φ_3_ = 180°, φ_4_ = 180°)** were provided in Supplementary Material for comparison (Figure S1).

The quantitative performance metrics extracted from these maps are summarized in Table 3, and the following observations are made when comparing across TMD channel configurations.

Considering |B_1_^+^| efficiency, the 4-CH TMD achieved the highest mean |B_1_^+^| efficiency among all TMD configurations (0.62 $\mu T/\sqrt{W}$), outperforming the 6-CH TMD (0.52 $\mu T/\sqrt{W}$) despite having fewer elements. This counterintuitive result is attributable to stronger inter-pair coupling in the 6-CH configuration, which degraded transmit efficiency. The 4-CH TMD also exhibited the highest peak |B_1_^+^| efficiency (1.9 $\mu T/\sqrt{W}$), though at the cost of moderate field uniformity, with a CV of 71%. The 2-CH TMD performed worst in terms of both mean efficiency (0.6 $\mu T/\sqrt{W}$) and homogeneity (CV = 80%).

Regarding SAR efficiency, the 6-CH TMD configuration achieved the highest mean SAR efficiency (1.2 $\mu T/\sqrt{W/kg}$), marginally outperforming the 4-CH configuration (1.1 $\mu T/\sqrt{W/kg}$) and substantially outperforming the 2-CH configuration (0.4 $\mu T/\sqrt{W/kg}$). The 4-CH configuration had the highest peak SAR efficiency (3.34 $\mu T/\sqrt{W/kg}$), driven by high surface fields rather than deep tissue penetration.

Regarding local $SAR_{10g}$, increasing the number of TMD channels resulted in substantially reduced mean and peak $SAR_{10g}$. The 6-CH configuration achieved the lowest mean $SAR_{10g}$ (0.1 W/kg), approximately half that of the 4-CH configuration (0.21 W/kg) and less than half that of the 2-CH configuration (0.24 W/kg). Peak $SAR_{10g}$ followed a similar trend, dropping from 0.66 W/kg in the 2-CH configuration to 0.27 W/kg in the 6-CH configuration, representing a 59% reduction.

**Figure 10:**
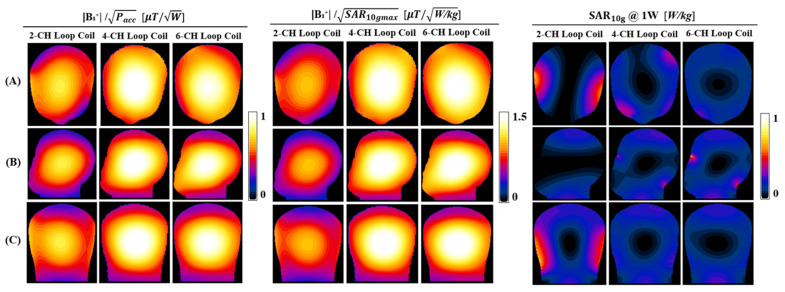
Simulated RF-optimized |B_1_^+^| efficiency (**left**), SAR efficiency (**middle**), and $SAR_{10g}$ maps at 1W $P_{acc}$ (**right**) in the (**A**) axial, (**B**) sagittal, and (**C**) coronal sections at isocenter of the head model for the two-, four-, and six-channel loop-coil configurations (Figure 3A–C). The respective color scales are identical to those in Figure 9 for comparison purposes.

Following the TMD results, the same analysis was performed for the loop-coil configuration in two-, four- and six-channel setups. The optimized RF shim phase settings for the loop coils were **[2-CH: φ_1_ = 158°, φ_2_ = 147°]**, **[4-CH: φ_1_ = 160°, φ_2_ = 234°, φ_3_ = 168°, φ_4_ = 250°]** and **[6-CH: φ_1_ = 40°, φ_2_ = 270°, φ_3_ = 195°, φ_4_ =56°, φ_5_ = 110°, φ_6_ = 5°]**, with amplitudes normalized identically to the TMD configurations as described in Section 2.2 (vi). The resulting |B_1_^+^| efficiency, SAR efficiency, and $SAR_{10g}$ maps are presented in Figure 10A–C for the same three sections of the head model at isocenter.

Similar to the TMD analysis, the quantitative performance metrics for the loop-coil arrays are summarized in Table 3, and the following observations are drawn when comparing across channel configurations.

Concerning |B_1_^+^| efficiency, the 2-CH loop coil achieved the lowest mean |B_1_^+^| efficiency (0.61 $\mu T/\sqrt{W}$), whereas the 4-CH (0.8 $\mu T/\sqrt{W}$) and 6-CH (0.73 $\mu T/\sqrt{W}$) configurations performed similarly, with the 4-CH configuration marginally superior. Peak |B_1_^+^| efficiency was highest for the 4-CH configuration (1.06 $\mu T/\sqrt{W}$), though differences across channel counts were modest. Field homogeneity was consistently good across all loop-coil configurations, with CV values of 28%, 23%, and 23% for 2-CH, 4-CH, and 6-CH configurations respectively, with 4-CH and 6-CH showing equivalence on this metric.

Regarding SAR efficiency, mean SAR efficiency improved substantially from 2-CH (0.8 $\mu T/\sqrt{W/kg}$) to 4-CH (1.2 $\mu T/\sqrt{W/kg}$) configurations but showed a marginal decrease for the 6-CH configuration (1.16 $\mu T/\sqrt{W/kg}$), suggesting diminishing returns beyond four channels, based on this metric. Peak SAR efficiency showed a similar pattern (1.11, 1.6, and 1.57 $\mu T/\sqrt{W/kg}$ for 2-CH, 4-CH, and 6-CH configurations, respectively).

Regarding local $SAR_{10g}$, the 2-CH loop coil produced the highest mean (0.3 W/kg) and peak (0.95 W/kg) $SAR_{10g}$ among all configurations of both array types. Increasing the channel count reduced mean $SAR_{10g}$ from 0.3 W/kg (2-CH) to 0.25 W/kg (4-CH) and 0.2 W/kg (6-CH), representing a 33% overall reduction. However, peak $SAR_{10g}$ did not follow the same trend, decreasing from 0.95 W/kg (2-CH) to 0.75 W/kg (4-CH) but rising again to 0.9 W/kg for the 6-CH configuration. This suggests that, although higher channel counts distributed the overall SAR more evenly, the increased complexity of the excitation pattern in larger arrays could introduce localized hotspots, potentially offsetting the benefit of reduced mean SAR.

The ${BW}_{-3dB}$ measurements for the loaded and unloaded conditions of all TMD and loop-coil configurations are summarized in Supplementary Material (Table S1). For the 2-CH TMD configuration, the bandwidth could not be reliably determined due to insufficient |S_11_| depth at the fundamental resonance, as discussed in Section 3.2.2. For the 4-CH and 6-CH TMD configurations, the loaded ${BW}_{-3dB}$ ranged from 2.1 MHz to 7 MHz. For the loop-coil arrays, the loaded ${BW}_{-3dB}$ ranged from 2.6 to 10.25, with the ratio decreasing as the number of channels increased.

**Figure 11:**
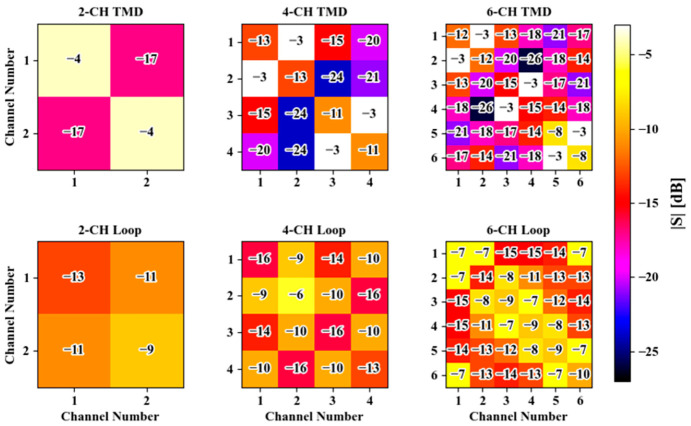
Simulated S-parameter matrices for the two-, four-, and six-channel head-loaded TMD arrays (**top row**) and loop-coil arrays (**bottom row**). All values are expressed as magnitude in dB.

The simulated S-parameter matrices for the TMD arrays (top row) and loop coil arrays (bottom row) are shown in Figure 11, illustrating the inter-element coupling and reflection coefficients across all channel configurations.

Having examined each array type independently, a direct comparison between the TMD and loop-coil configurations across matched channel counts reveals the following observations.

**B_1_^+^ Efficiency:** Regarding mean |B_1_^+^| efficiency, the loop coil arrays consistently outperformed the TMD arrays across all channel configurations. The advantage was least pronounced at the 2-CH level, where the loop coil (0.61 $\mu T/\sqrt{W}$) and TMD (0.60 $\mu T/\sqrt{W}$) were nearly equivalent. However, the effect was substantial for both 4-CH configurations (loop: 0.8 vs. TMD: 0.62 $\mu T/\sqrt{W}$) and 6-CH configurations (loop: 0.73 vs. TMD: 0.52 $\mu T/\sqrt{W}$)—constituting advantages of 29% and 40% for loop coils, respectively.

Additionally, TMD arrays achieved substantially higher peak |B_1_^+^| efficiency values than loop coils across all configurations: 47% higher for 2-CH (1.54 vs. 0.82 $\mu T/\sqrt{W}$), 79% higher for 4-CH (1.9 vs. 1.06 $\mu T/\sqrt{W}$), and 33% higher for 6-CH (1.3 vs. 0.98 $\mu T/\sqrt{W}$). As evident from the B1^+^ maps, however, the high peak values for the TMD arrays were concentrated in regions close to the dipole feed and along the α line rather than deep within the tissue (as for the loop arrays), reducing deep-tissue imaging capability.

In further observations, the loop-coil arrays showed markedly superior |B_1_^+^| homogeneity throughout the head, across all channel counts, with CV values two to three times lower than those of TMD arrays (e.g., 4-CH: loop CV = 23% vs. TMD CV = 71%; 6-CH: loop CV = 23% vs. TMD CV = 53%). Notably, both configurations showed limited |B_1_^+^| transmission coverage at the top and bottom of the head. Coverage was slightly less for the TMD array than for the loop-coil configurations.

**SAR Efficiency:** For mean SAR efficiency, the loop coil outperformed the TMD array significantly in the 2-CH configuration (0.8 vs. 0.4 $\mu T/\sqrt{W/kg}$, a 2× advantage), whereas both were effectively equivalent at 4-CH (1.2 vs. 1.1 $\mu T/\sqrt{W/kg}$) and 6-CH (1.16 vs. 1.2 $\mu T/\sqrt{W/kg}$). Peak SAR efficiency was also similar for loop and TMD arrays at 2-CH (1.1 vs. 1.08 $\mu T/\sqrt{W/kg}$) but was higher for TMD arrays at 4-CH (TMD: 3.34 vs. loop: 1.6 $\mu T/\sqrt{W/kg}$) and at 6-CH (TMD: 2.9 vs. loop: 1.57 $\mu T/\sqrt{W/kg}$). The latter two comparisons reflect the surface-concentrated B_1_^+^ field and low local $SAR_{10g}$ characteristics of the TMD arrays (see immediately below).

**Local** $SAR_{10g}$**:** Mean local $SAR_{10g}$ was generally lower for TMD arrays than for loop coil arrays across all configurations: 25% lower at 2-CH (TMD: 0.24 vs. loop: 0.3 W/kg), 16% lower at 4-CH (TMD: 0.21 vs. loop: 0.25 W/kg), and 50% lower at 6-CH (TMD: 0.1 vs. loop: 0.2 W/kg). Peak local $SAR_{10g}$ further confirmed this advantage. TMD arrays produced substantially lower peak values than loop coil arrays across all configurations: 44% lower at 2-CH (TMD: 0.66 vs. loop: 0.95 W/kg), 26% lower at 4-CH (TMD: 0.56 vs. loop: 0.75 W/kg), and 70% lower at 6-CH (TMD: 0.27 vs. loop: 0.9 W/kg). These results suggest a possible safety advantage for TMD arrays in terms of local power deposition, for the 4-CH and 6-CH configurations. For additional context, both array types showed pronounced $SAR_{10g}$ “cool spots” of low power deposition within the head across all channel configurations. However, some “warm spots” of elevated power were also apparent near the neck and temporal regions in the TMD arrays (mainly for the 2CH configuration) and in the eye and posterior head regions in the loop-coil arrays (across all channel counts and with more focality and intensity for the 4-CH and 6-CH configurations).

Additionally, supplementary simulations are presented in Figures S2 and S3. Figure S2 shows the |B_1_^+^| efficiency, SAR efficiency, and $SAR_{10g}$ maps for the birdcage coil loaded with the uniform head model. The birdcage coil driven in CP mode achieved marginally superior B_1_^+^ and SAR efficiency compared to the six-channel loop coil, with similar $SAR_{10g}$ values. Figure S3 presents the corresponding results for the four-channel TMD array simulated with the MIDA multi-tissue head model, which showed good agreement with the uniform head model results in Figure 9, confirming that the primary findings were minimally sensitive to the choice of head model.

### 3.3. Experimental Results

**Figure 12:**
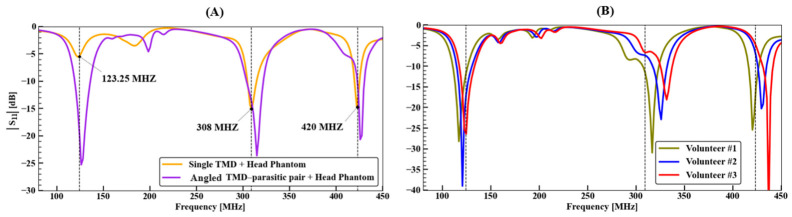
Benchtop |S_11_| measurements of TMD elements under different loading conditions. (**A**) Single CH1 element and angled CH1–CH2 TMD–parasitic pair with CH3 and CH4 inactive, loaded with a head phantom. Similar behavior was observed for CH3 and the CH3–CH4 pair. (**B**) Angled CH1–CH2 TMD–parasitic pair loaded separately with the heads of three volunteers. Dashed vertical lines in both panels indicate the three resonance peaks at approximately 123 MHz, 308 MHz, and 420 MHz, corresponding to the static magnetic fields for MRI at 3 T, 7 T, and 9 T, respectively.

Following the four-channel TMD array and head phantom setup described in Section 2.3 and shown in Figure 3E, the |S_11_| frequency response of CH1 was measured both alone and when paired with CH2 as a TMD–parasitic pair, with CH3 and CH4 inactive. Direct benchtop characterization of the driven TMD pair under simultaneous multi-channel excitation was not performed in the present study due to experimental constraints. The implications of this limitation and the associated measurement considerations are discussed in Section 4.4 below. The measured |S_11_| responses are shown in Figure 12A for the single CH1 and paired (angled) CH1–CH2 configurations, respectively, and were consistent with the simulation findings. A minor spurious peak in Figure 12 was attributed to residual coupling with the coaxial-cable baluns, and was not substantially affected by dipole pairing. Reducing the inter-element spacing in paired dipoles to less than 3 cm induced resonance peak splitting and corresponding S_11_ phase shifts.

Inter-element coupling between CH1 and CH2 in the paired configuration was ~−3 dB. Similar results were obtained for CH3 and the CH3–CH4 pair, confirming the simulation results. Inter-pair coupling between the opposing pairs, CH1–CH2 vs. CH3–CH4, was ≤−20 dB.

To assess frequency shift under in-vivo loading conditions, the angled CH1–CH2 TMD–parasitic pair was loaded with the heads of three female volunteers (age range: 34–45 years), and the |S_11_| responses are shown in Figure 12B. The results were consistent with the head phantom measurements, with minor frequency deviations observed at $f_{0}$ and progressively larger shifts at higher-order harmonics across volunteers. Variations in |S_11_| frequency response across volunteers are attributed to differences in head geometry, which alter the effective dipole-to-head distance. As demonstrated in Figure 5, dipole-to-head distance is the primary determinant of the observed frequency shifts rather than head size or shape. This effect is relatively minor and can be mitigated by applying a small angular adjustment to the dipole assembly, such that one arm is positioned slightly closer to the head surface and the other slightly farther depending on patient head geometry.

**Figure 13:**
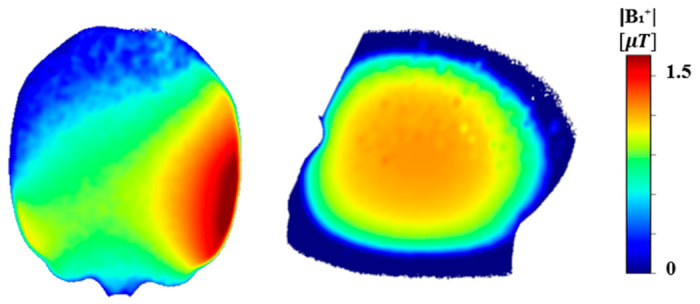
Experimentally measured B_1_^+^ maps of the head phantom acquired with the 4-channel TMD array using the double angle method (DAM) with 30° and 60° flip angles, at the axial and sagittal isocenters. The small areas of signal loss observed within the phantom are due to air bubbles that were trapped during the general preparation. The pronounced dropout at the top left of the sagittal section is due to incomplete phantom filling, with air migrating toward the forehead upon horizontal positioning.

Subsequently, GRE imaging of the configuration shown in Figure 3E (i.e., two driven TMD pairs) showed no temperature rise at any of the three sensors, supporting in-vitro thermal safety for head MRI. Imaging was conducted using the transmit phase settings obtained from the simulated 4-channel optimization (φ_1_ = 0°, φ_2_ = 0°, φ_3_ = 180°, φ_4_ = 250°). The experimental B_1_^+^ maps acquired at the axial and sagittal isocenters using the DAM are shown in Figure 13, providing direct quantitative evaluation of the transmit field homogeneity achieved with the four-channel TMD array. The measured |B_1_^+^| distributions showed reasonable agreement with the simulated |B_1_^+^| efficiency maps in Figure 9.

## 4. Discussion

Through careful, sequential progression of proof-of-concept simulations and experiments, the present work indicates the potential of TMD transmit arrays for MRI of the head and brain at 3 T. The results demonstrate the feasibility of TMD-based transmission, supported by MRI data for a head phantom, with simulations suggesting that SAR in human use is expected to remain low. However, additional work is needed to address limitations of the present study and to translate the TMD concept into a practical architecture for clinical MRI applications. In the discussion that follows, the narrative is organized to follow the sequence of Section 3, emphasizing interpretation of the underlying physical mechanisms and the novelty of the observed behaviors, and ending with the significance of the work, the associated limitations, and directions for future investigation.

### 4.1. Initial Experimental Observations: Interpretation

The six key observations reported in the Results Section (Section 3.1) were confirmed by simulations. In the following, each observation is examined in detail, with emphasis on the underlying EM mechanisms, implications for dipole design in 3 T MRI applications, and potential strategies for optimizing performance in multi-channel arrays.

### 4.2. Simulation Results: Interpretation and Significance

#### 4.2.1. Step (i): Frequency Response of Five Unloaded Dipole Geometries

**Figure 14:**
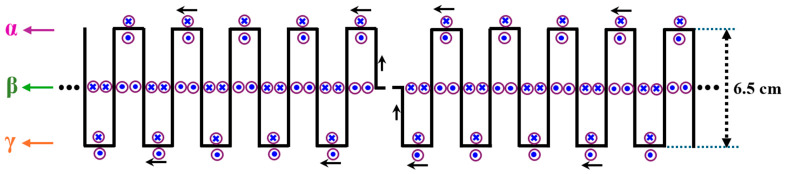
Schematic of the TMD used in all simulations and experiments in the present study. Black arrows indicate the direction of current flow along the dipole at one instant in time, whereas blue dots and crosses represent the B_1_^+^ field pointing out of and into the page, respectively.

The frequency-response behavior observed in the initial benchtop experiments and depicted by simulations in Section 3.2.1 uncovered several key findings. First, for the geometries studied, the transformation from the SD to the TMD resulted in an increase in the fundamental resonance frequency $\left(f_{0}\to f_{0}+\Delta f\right)$, representing a key novel observation. This shift arises from EM coupling between intra-meander segments within a single dipole, which effectively shortens the $L_{e}\;$ value of the dipole. As indicated schematically in Figure 14 using B_1_^+^ vector fields, the dominant current mode at the fundamental resonance exhibits fields that are highly prone to cancelation within the meander structure. Considering the vector fields close to the vertical segments at the β-line, it is evident that meanders produce a series of alternating clockwise and counterclockwise fields, which tend to cancel each other given that the meanders are tightly spaced. The cancellation effect is broken near the feed point where the elongated parallel-conductor geometry is absent; and is weakened with increasing vertical distance toward the α- and γ- lines as the superposition of B_1_^+^ fields from the horizontal sections of the meanders becomes more influential. These effects are readily observed in the |B_1_^+^| map of the TMD shown in Figure 6A. Furthermore, the partial cancelation effect indicates that a portion of the current-carrying length contributes weakly to the resonance, resulting in a reduced effective electrical wavelength (i.e., λ_0_ → λ_0_ − Δλ) and increase in the fundamental resonance frequency from that of the SD geometry.

Consistent with the observation above, successive resonant harmonics no longer occurred at integer multiples of the fundamental frequency and became progressively closer spaced. This behavior follows from the electrically shortened current path at the fundamental mode, as well as imperfect field cancelation in the alternating vertical segments and the segmented nature of the horizontal conductors. Consequently, higher-order resonances appear at frequencies lower than the integer multiples of $f_{0}$, reflecting the complex distribution of currents in the TMD geometry.

Additionally, |S_11_| was observed to decrease from NAZD to TMD across all harmonics, while remaining nearly constant for less compact geometries. This behavior is consistent with increased partial magnetic-field cancelation along the dipole midline as the meander density increases. Stronger intra-meander coupling and opposing field directions reduce the effective radiating current, leading to poorer power transfer from the feed. Furthermore, increasing $L_{c}$ length, independent of $L_{p}$ length, shifts the resonance peaks to lower frequencies due to the inverse relationship between frequency and wavelength.

#### 4.2.2. Step (ii): Frequency Response of a TMD Loaded with the Head Model

As demonstrated in Section 3.2.2, all resonance peaks shifted toward lower frequencies upon loading, with the shift magnitude increasing as the separation between the TMD and the head model decreased (Figure 5). The fundamental resonance decreased by approximately 23 MHz at a 5 mm separation, from 146 MHz in the unloaded case (Figure 4E) to 123 MHz under loading (Figure 5). This loading-induced downward shift is consistent with previous reports for UHF dipoles used in high-field MRI, where loading similarly reduces the resonance frequency [45]. The high sensitivity to loading can be partially mitigated by the relatively broad bandwidth of the TMD, although this may remain a challenge for MRI of the head.

#### 4.2.3. Step (iii): Near-Field Evaluation of a TMD in Free Space and Inside the Head Medium

As shown in Section 3.2.3, reducing *L_w_* from 6.5 cm to 1 cm (at fixed *L_p_*length) improved |B_1_^+^| uniformity at 298 MHz within the medium. However, as noted in the Results, achieving 128 MHz would require thinner traces, imposing practical current-handling and heating constraints. This underscores a trade-off between miniaturization and safe power handling in TMD design for MRI applications.

#### 4.2.4. Step (iv): Inter-Element Interaction of a Coplanar TMD Pair

For both the coplanar TMD–parasitic pair and the coplanar driven TMD pair, in free space and under loading (Section 3.2.4), |S_11_| increased relative to a single TMD element. As the inter-element separation decreased, |S_11_| increased. At a separation of approximately 3 cm, the magnitude of |S_11_| increased by at least a factor of three compared with the single-element case.

At separations smaller than approximately 3 cm, inter-element coupling-induced modifications of the resonance behavior such as splitting of the primary resonance into two distinct modes, are expected to become pronounced. In the present study, a minimum TMD-to-TMD separation of ~3 cm was maintained, so such splitting was not observed.

For higher-channel TMD arrays (e.g., eight channels or more), where inter-element spacing may be constrained, similar coupling effects are expected. These coupling effects can be mitigated by slight detuning of paired elements, adjusting the termination resistance in the TMD–parasitic configuration, or optimizing the relative phase offsets in the driven TMD configuration; ultimately to reduce destructive |B_1_^+^| interference and help preserve SNR.

#### 4.2.5. Step (v): Directionality

Two dipole configurations were analyzed in Section 3.2.5—a driven TMD paired with a parasitic element and a driven TMD pair—using simulated Poynting vector maps (Figure 8A,B). In free space with a coplanar arrangement, the driven TMD–parasitic pair produced an asymmetric power-flow pattern, directing energy away from the parasitic element, whereas the coplanar driven TMD pair maintained a symmetric pattern. Under head loading (with TMD pair elements angled slightly to conform with the head surface), both configurations exhibit some power reflection, but the driven TMD pair delivers energy slightly more efficiently due to its symmetric excitation, which reduces destructive interference and directs more power toward the target. The driven TMD pair was therefore selected for all subsequent array simulations. The TMD–parasitic pair configuration may nonetheless be of interest in setups where the number of available transmit channels is limited, such as a two-transmit/four-receive architecture. The use of additional reflectors—such as high-permittivity dielectric pads [46]—could potentially improve energy delivery, but the effectiveness of this strategy depends strongly on shape, size, and permittivity, and remains an open area for further investigation.

#### 4.2.6. Step (vi): SAR and |B_1_^+^| Assessment of TMD vs. Loop Arrays

The optimized |B_1_^+^| efficiency, SAR efficiency, and corresponding $SAR_{10g}$ maps for the two-, four-, and six-channel TMD arrays (Figure 9) and loop-coil arrays (Figure 10) from Section 3.2.6 show differences between the array types. The loop-coil arrays achieve higher |B_1_^+^| efficiency with more uniform coverage throughout the head volume, whereas the TMD arrays produce substantially higher |B_1_^+^| efficiency near the head surface. As a result, achieving a target flip angle at depth with TMD arrays requires higher transmit power, leading to increased surface excitation and elevated local SAR. By comparison, loop-coil arrays maintain a more uniform excitation profile across depth. This characteristic of the TMD is primarily attributed to two factors. First, the TMDs are necessarily placed in close proximity to the head (0.5 cm), whereas the loop coils are positioned farther away (≥2 cm from the head model). Close placement of the TMDs results in strong surface |B_1_^+^| excitation, whereas more remote placement reduces the penetration of these high-intensity fields into the medium. Second, as per Figure 14, |B_1_^+^| contributions from the counter-rotating α- and γ-line sections partially cancel as the distance from the TMD increases, further reducing |B_1_^+^| field as a function of depth into the head.

Furthermore, the optimized phase distributions in the TMD arrays reveal a consistent pairing-dependent behavior. Within each pair, the phase differences between neighboring elements remain relatively small compared to those observed between different pairs, indicating a quasi-linear excitation at the pair level. In contrast, the phase relationships between pairs follow a pattern broadly consistent with a circularly polarized (CP-like) excitation across the array. This structure is observed across all configurations: in the 2-CH configuration (φ_1_ = 0°, φ_2_ = 180°), the two elements are driven in antiphase, consistent with CP mode; in the 4-CH configuration, φ_1_ = 0° and φ_2_ = 0° form pair 1, while φ_3_ = 180° and φ_4_ = 250° form pair 2; and in the 6-CH configuration, φ_1_ = 0°, φ_2_ = 0°, φ_3_ = 120°, φ_4_ = 120°, φ_5_ = 240°, and φ_6_ = 240° define the three pairs, with inter-pair phase offsets of approximately 120° consistent with a three-phase CP-like excitation pattern.

Additionally, the central lateral SAR null observed within the head for the two-, four-, and six-channel TMD arrays persists across different RF shimming solutions. This pattern arises from the predominantly perpendicular power flow of dipole elements relative to their axis, resulting in laterally directed field penetration. For the loop-coil geometries, however, the location of the SAR null is less constrained and can appear in different orientations depending on the input phase settings of the array elements. This behavior reflects the intrinsic field distribution of individual loop coils, which exhibit a central SAR null and generate fields primarily along the conductor circumference. Furthermore, loop-coil geometries may exhibit locally elevated power deposition, particularly in the vicinity of tuning capacitors [47]. Although the precise characteristics of the power deposition in these regions could be affected by meshing accuracy in the EM simulations (e.g., for the eye and posterior head regions), the overall trend remains evident.

### 4.3. Significance of the Work

The present work makes several contributions to the field of RF coil design for MRI. First, previous studies on meandered dipoles—primarily conducted in the telecommunications field—either varied the number of meanders while keeping the dipole $L_{p}$ length fixed [45,48,49,50] or shortened $L_{p}$ by increasing the number of meanders while keeping the $L_{c}$ length constant [50]. The present work, for the first time, investigates the continuous transformation of an SD into a TMD while maintaining a fixed number of meanders (fixed $L_{c}$), revealing that progressive compaction shifts the resonance frequency profile upward and reduces |S_11_|, as discussed in Section 4.2.1. Furthermore, both the inter-meander spacing and the separation between the α and γ lines were found to strongly govern the EM behavior of the structure. Reducing the inter-meander spacing leads to coupling between adjacent horizontal segments, as reflected in the H-shaped near-field pattern in Figure 6. To the best of our knowledge, the role of the α–γ line separation in determining the EM behavior of the meandered dipole has not been previously reported. Together with the transformation study in Section 3.2, these findings provide a practical framework and additional insight for compact dipole design in MRI at 3 T and higher magnetic fields.

Second, although meandered dipoles have been explored in MRI, studies at 3 T remain limited to prostate imaging [34,35] without systematic investigation of geometry, resonance behavior, or array performance. Careful consideration of TMD arrays for 3 T MRI of the head is thus a novel and useful contribution. Moreover, unlike prior implementations that relied on lumped-element tuning networks, resonance was controlled solely through conductor geometry in the present work, enabling a more predictable design approach.

Third, rather than treating inter-element coupling as an undesirable effect to be suppressed, the present work deliberately exploited interaction between elements within a pair at a defined spacing to substantially increase transmit gain compared to a single unpaired element. This approach is conceptually distinct from both fractionated dipole arrays and conventional loop coil arrays. Inter-pair coupling, however, remains an undesirable effect that needs to be addressed as the number of channels increases.

Fourth, the comparison between TMD and loop coil arrays across two-, four-, and six-channel configurations provides a quantitative benchmark of dipole-based transmit performance against the established loop coil standard at 3 T. Despite lower mean |B_1_^+^| efficiency in depth compared to loop coil arrays, the TMD arrays demonstrate SAR performance which makes them potentially attractive for channel counts of 4 and above (whereas the 2-CH TMD configuration showed low SAR efficiency, making it impractical). Notably, both mean and peak local $SAR_{10g}$ were consistently lower for the TMD arrays compared to loop coils for 4-CH and 6-CH configurations, with peak $SAR_{10g}$ reductions of 26%, and 70% at 4-CH, and 6-CH respectively compared to the loop coil arrays, confirming a meaningful safety advantage in terms of local energy deposition, particularly in the peripheral regions of the head.

### 4.4. Limitations of TMD Array

The TMD design eliminates the need for tuning and matching circuits, allowing resonance adjustment via dipole length or dipole–patient spacing. The EM response, however, is highly sensitive to loading conditions. The broader resonance bandwidth of the TMDs compared with the loop coil (as summarized in Table S1, with head-loaded ${BW}_{-3dB}$ ranging from 2.1 to 7 MHz for the 4CH and 6-CH driven TMDs versus 2.6 to 5.8 MHz for the corresponding loop-coil configurations in the present work) partially mitigates this effect by reducing detuning under varying loading conditions. However, loading sensitivity remains an issue that should be investigated thoroughly in patients—including the possibility of dynamic sensitivity variations in the presence of patient motion, which require careful adoption of MRI methods to reduce motion artifacts.

Simulated RF shim maps (Figure 9) show signal voids at the head top and lower neck, corresponding to regions distant from the TMD active segments where |B_1_^+^| decays along the longitudinal axis. Additionally, larger separations between the TMD elements and the patient exacerbate partial field cancelation between counter-rotating segments, reducing |B_1_^+^| efficiency. Future TMD designs should consider the need to increase volume of coverage for practical head imaging, possibly with increased focus on careful patient positioning to place anatomical regions of clinical interest appropriately within the field of view.

Benchtop characterization of the driven TMD pair was further limited by the available measurement setup. Specifically, simultaneous excitation of multiple channels with controlled phase relationships was not feasible using the single-port network analyzer available in the present work. As a result, S-parameter measurements were performed on individual channels or passive configurations only.

Furthermore, the Q ratio (Q_unloaded_/Q_loaded_) could not be reliably calculated for the TMD geometry for two reasons. First, loading the TMD with the head phantom produces a substantial decrease in resonance frequency, from approximately 146 MHz in free space to 123.25 MHz under loading, such that the unloaded and loaded Q factors correspond to fundamentally different frequencies. Second, loop coils are primarily loaded by the electrical resistance of the patient with commonly near-zero frequency shift, whereas dipole antenna design traditionally focuses on the radiation of energy into free space. Changes in dipole Q values between unloaded and loaded conditions reflect a combination of sample loading and radiation losses rather than sample coupling alone [51,52], making the conventional Q ratio interpretation less meaningful for dipole-based designs. To address this, some studies have proposed measuring Q_shielded_ in place of Q_unloaded_ [52]; however, the appropriate shield geometry for the TMD configuration requires dedicated investigation and is beyond the scope of the present work. The ${BW}_{-3dB}$ and loaded/unloaded ${BW}_{-3dB}$ ratio are reported in Table S1 for both TMD and loop-coil geometries. It should be noted, however, that the BW ratio values for the TMD are all less than one, which is physically unrealistic for a sample-loaded resonator and further confirms that the unloaded ${BW}_{-3dB}$ of the TMD is not representative of true coil loss due to the dominance of radiation losses in free space, and should therefore be interpreted with caution. The Poynting vector maps in Figure 8 are additionally used as the more physically appropriate characterization of energy deposition for the loaded vs. unloaded TMD configurations.

In the current simulation geometry and computing framework, the UPML did not fully suppress boundary reflections, producing numerical instabilities that affected the dipole response in some cases. These effects appeared as oscillations in the |S_11_| reflection coefficient and spurious resonance peaks (see Figure 7A, for example), particularly when increasing folding from NAZD to TMD configurations or when the TMD width $L_{w}$ exceeded 3 cm. These spurious resonances for the 6.5 cm TMD geometry occurred at frequencies (255, 413, and 463 MHz) well above the operating frequency (123.25 MHz) and therefore did not affect the computed EM fields at the frequency of interest. The minor nature of the errors is supported by the strong agreement between simulated and benchtop measured $S_{11}$ at 123.25 MHz, confirming the reliability of the field solutions at the operating frequency. In addition, time and spatial discretization in the simulation setups were chosen to delay boundary reflections and reduce their influence. This approach minimized numerical artifacts, and further improvements in FDTD boundary treatments for such compact meandered geometries are beyond the scope of the present work.

### 4.5. Potential Applications and Future Work

Several directions are currently under exploration to address the limitations identified above. Positioning the brain closer to the isocenter of the array through careful design of the coil housing and mounting apparatus would reduce signal voids at the top of the head. Dipole design modifications may improve |S_11_| values without the need for pairing TMD-to-TMD elements, such as introducing asymmetric, non-equal meanders applied to both arms; using alternative geometries like NAZD, which involve a trade-off between the *L_p_* and *L_w_* values of the dipole and the distance to the patient; or a combination of both approaches. Whole-head uniformity could be further improved by adding channels (e.g., eight channels) with feed points distributed across the head. Multi-channel excitation and phase-controlled measurements using multi-port vector network analyzers or dedicated phased-array measurement systems remained an important area for future experimental validation and optimization.

Additionally, the use of a ground plane in a microstrip-like configuration [30,31] may offer improvements in penetration depth, SNR, and inter-pair coupling. Preliminary experimentation with this approach introduced additional dependence on substrate characteristics and ground plane geometry, making the resonance behavior less predictable compared to the geometry-controlled TMD design. Whether such structures can be integrated into TMDs without compromising the simplicity and predictability of the geometry-based tuning approach remains an open question for future investigation.

An interesting future direction is the extension of TMD arrays to multinuclear imaging and operation at other static magnetic field values, including ultra-high field MRI. Such work would take advantage of the inherently multi-resonant characteristics of TMDs, either by exploiting harmonic resonances or through targeted modifications to the conductor geometry such as adjusting the number of meanders, or a combination of both. As part of such work or as part of the refinements indicated above, TMDs can be readily fabricated using printed circuit board (PCB) technology and integrated with programmable switching components, enabling resonance tuning to be adjusted remotely at the console by the operator prior to imaging. The substantially lower local $SAR_{10g}$ demonstrated by the TMD configuration compared to loop-coil configuration suggests that further exploration is warranted, for example in 3 T MRI applications involving patients with electrically conductive medical implants that are prone to RF heating. Finally, the open and flexible structural design of TMDs allows for placement at different locations around the patient target anatomy and facilitates adaptation to a range of clinical MRI applications at 3 T and higher fields, for example involving the extremities such as the hands and legs, in situations where SAR concerns are substantial.

## Figures and Tables

**Table 1:** Q factors of fundamental and four higher harmonic resonances for the five unloaded dipole geometries shown in Figure 2A. $f_{n-1}$ denotes the *n*th harmonic.

	SD	WAZD	MAZD	NAZD	TMD
$f_{0}$	45	46	32	14.3	-
$f_{1}$	27	282	41	-	-
$f_{2}$	38.5	117.5	76.2	-	-
$f_{3}$	40	40	69	-	-
$f_{4}$	41.7	52.3	47	-	-

**Table 2:** Q-factors of the fundamental and next two higher harmonic resonances for the simulated head-loaded TMD–parasitic and driven TMD pair configurations. $f_{n-1}$ denotes the *n*th harmonic.

	Q Factor for Loaded TMD–Parasitic Pair	Q Factor for Loaded Driven TMD Pair
$f_{0}$	50.6	25.7
$f_{1}$	74.2	76.3
$f_{2}$	63.3	90

**Table 3:** Summary of transmit performance metrics for the two-, four-, and six-channel TMD and loop-coil arrays. Mean and peak values are calculated from the |B_1_^+^| efficiency, SAR efficiency, and $SAR_{10g}$ maps shown in Figure 9 and Figure 10, respectively. The coefficient of variation (CV) quantifies the spatial homogeneity of |B_1_^+^| efficiency within the entire head.

	Unit	TMD			Loop Coil		
		2-CH	4-CH	6-CH	2-CH	4-CH	6-CH
**B_1_^+^ Efficiency**							
Mean B_1_^+^ efficiency	$\mu T/\sqrt{W}$	0.6	0.62	0.52	0.61	0.8	0.73
Peak B_1_^+^ efficiency	$\mu T/\sqrt{W}$	1.54	1.9	1.3	0.82	1.06	0.98
B_1_^+^ efficiency CV	%	80	71	53	28	23	23
**SAR Efficiency**							
Mean SAR efficiency	$\mu T/\sqrt{W/kg}$	0.4	1.1	1.2	0.8	1.2	1.16
Peak SAR efficiency	$\mu T/\sqrt{W/kg}$	1.08	3.34	2.9	1.11	1.6	1.57
**Local** $SAR_{10g}$							
Mean $SAR_{10g}$	W/kg	0.24	0.21	0.1	0.3	0.25	0.2
Peak $SAR_{10g}$	W/kg	0.66	0.56	0.27	0.95	0.75	0.9

## Data Availability

The data presented are available on request from the corresponding authors.

## Supplementary Materials

The following supporting information can be downloaded at: https://www.mdpi.com/article/10.3390/s26123867/s1, Figure S1: CP-mode |B_1_^+^| efficiency, SAR efficiency, and $SAR_{10g}$ maps for the four-channel TMD array. Figure S2: CP-mode |B_1_^+^| efficiency, SAR efficiency, and $SAR_{10g}$ maps for a birdcage coil loaded with the uniform head model. Figure S3: |B_1_^+^| efficiency, SAR efficiency, and $SAR_{10g}$ maps for the four-channel TMD array with the MIDA (Multimodal Imaging-Based Detailed Anatomical) head model. Table S1. Measured −3 dB bandwidth (${BW}_{-3dB}$) in loaded and unloaded conditions for all TMD and loop-coil configurations.

## Abbreviations

The following abbreviations are used in this manuscript:

MRI	Magnetic resonance imaging
SAR	Specific absorption rate
RF	Radiofrequency
pTx	Parallel radiofrequency transmission
ROI	Region of interest
UHF	Ultra-high field
T	Tesla
MHz	Mega hertz
EM	Electromagnetic
SD	Straight dipole
WAZD	Wide-angle zigzag dipole
MAZD	Moderate-angle zigzag dipole
NAZD	Narrow-angle zigzag dipole
TMD	Tightly meandered dipole
FDTD	Finite-difference time-domain
UPML	Uniaxial perfectly matched layer
GRE	Gradient-echo
