# Supplementary material for: Detailed Consideration of a Novel Meandered Dipole Array for Magnetic Resonance Imaging of the Head at 3 Tesla with Low Radiofrequency Power Deposition
Source: Sensors (Basel). 2026 Jun 17;26(12):3867. doi: 10.3390/s26123867 (PMC13306261; doi:10.3390/s26123867)
Supplement: Supplementary file 1 [file sensors-26-03867-s001.zip › sensors-4294295-supplementary.pdf]

Supplementary Materials:

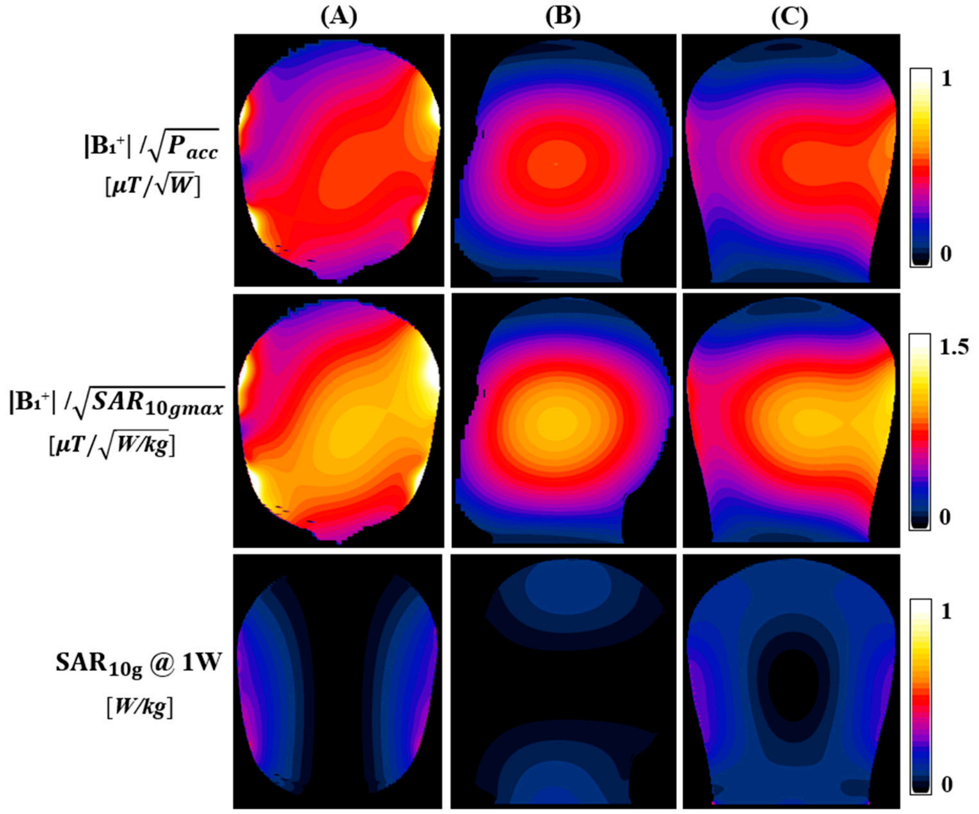

Figure S1: Simulated $|B_1^+|$ efficiency, SAR efficiency, and SAR_{10g} maps at 1 W P_{acc} in the **(A)** axial, **(B)** sagittal, and **(C)** coronal sections at isocenter, for the four-channel TMD array driven with CP-mode phase settings ($\varphi_1 = 0°$, $\varphi_2 = 0°$, $\varphi_3 = 180°$, $\varphi_4 = 180°$), shown for comparison with the RF-shimmed results in Figures 9A–C.

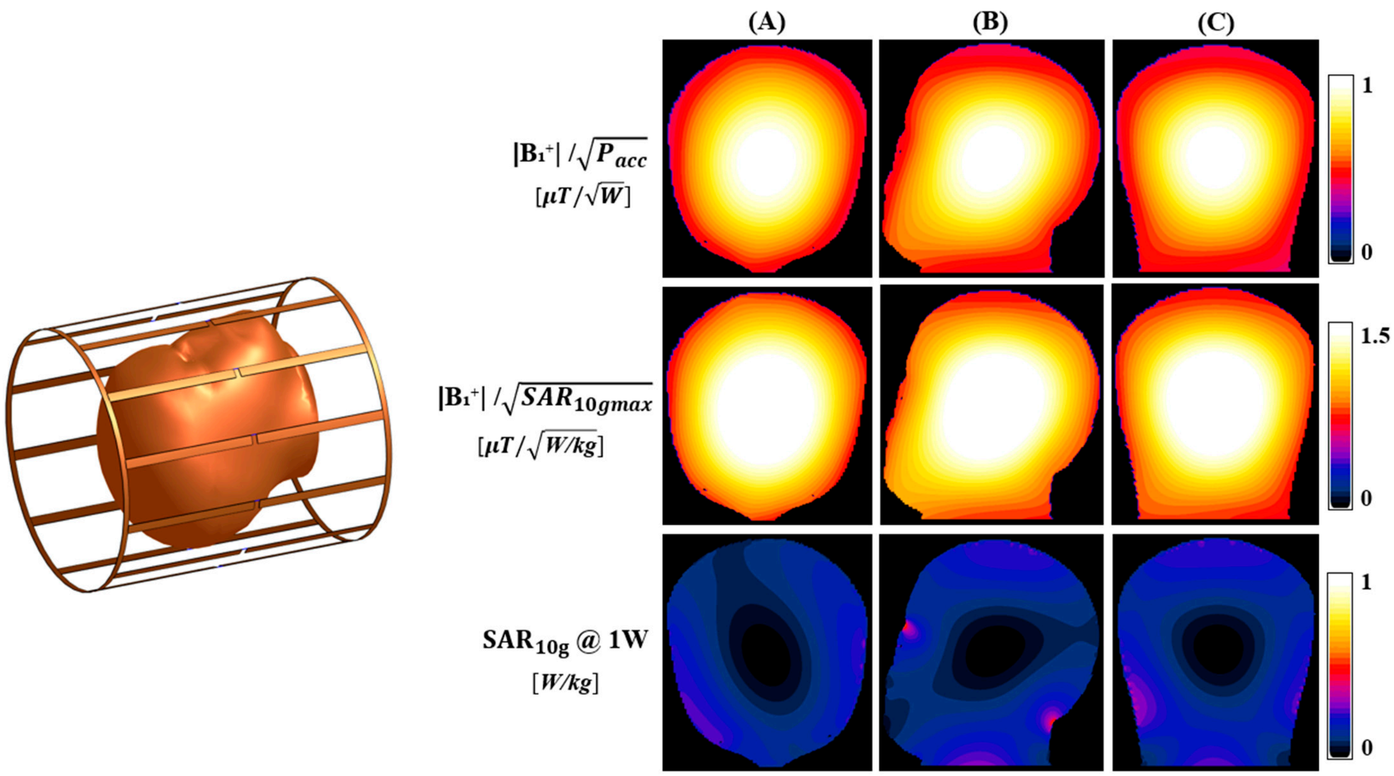

Figure S2: Simulated CP-mode $|B_1^+|$ efficiency, SAR efficiency, and SAR_{10g} maps at 1W P_{acc} in the **(A)** axial, **(B)** sagittal, and **(C)** coronal sections at isocenter, for a birdcage coil loaded with the uniform head model. The simulation geometry is shown in the left-most panel. The birdcage coil was driven in quadrature mode with φ_1 = 0° and φ_2 = 90°, yielding $|S_{11}|$ = −17 dB, $|S_{22}|$ = −13 dB, and $|S_{12}|$ = $|S_{21}|$ = −22 dB. The normalization procedure follows the same approach as described for the TMD and loop-coil arrays in Figures 9 and 10, enabling direct comparison across all three coil configurations.

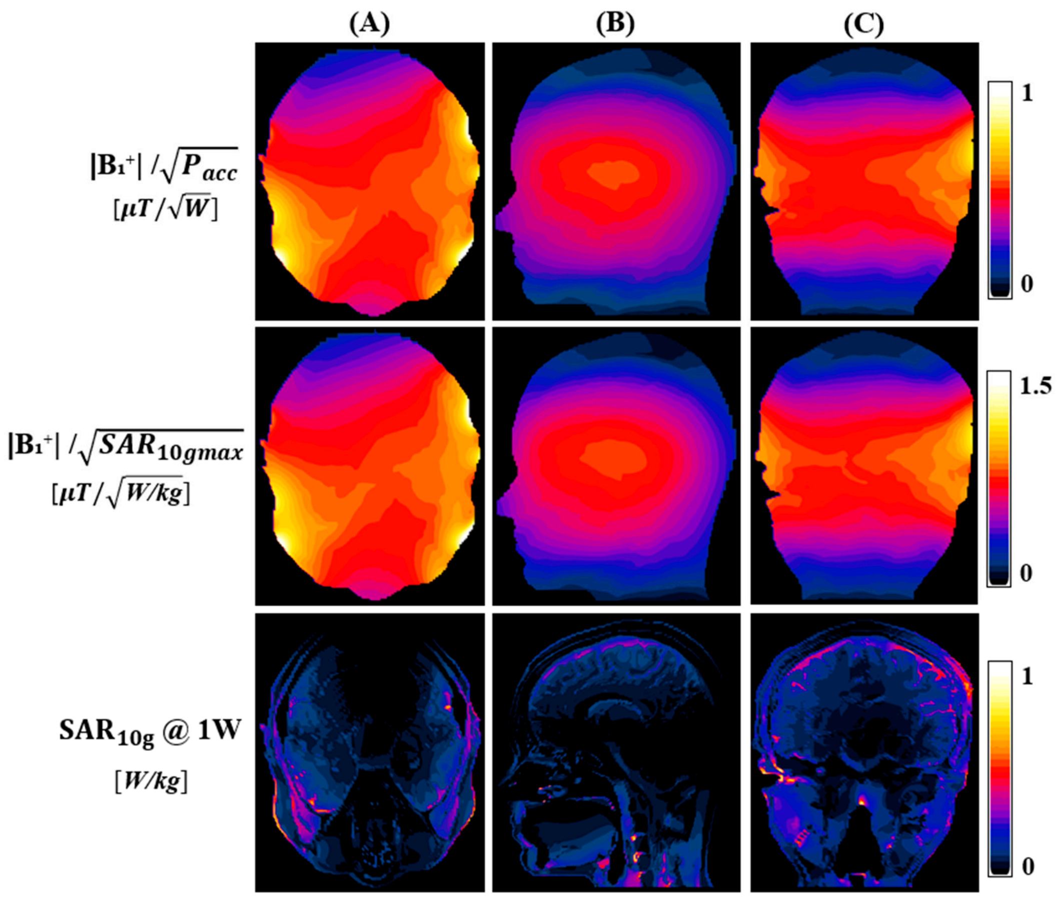

Figure S3: Simulated RF-shimmed $|B_1^+|$ efficiency, SAR efficiency, and SAR_{10g} maps at 1W P_{acc} in the **(A)** axial, **(B)** sagittal, and **(C)** coronal sections at isocenter, for the four-channel TMD array simulated with the MIDA (Multimodal Imaging-Based Detailed Anatomical) head model. The array configuration, optimized phase settings, and normalization procedure are identical to those used in Figure 3B and Figure 9. The simulated S-parameters for the MIDA-loaded four-channel TMD array are: $|S_{11}|$ = −9 dB, $|S_{22}|$ = −11 dB, $|S_{33}|$ = −11 dB, $|S_{44}|$ = −11 dB; $|S_{12}|$ = $|S_{21}|$ = −4 dB, $|S_{13}|$ = $|S_{31}|$ = −19 dB, $|S_{14}|$ = $|S_{41}|$ = −18 dB, $|S_{23}|$ = $|S_{32}|$ = −18 dB, $|S_{24}|$ = $|S_{42}|$ = −16 dB, $|S_{34}|$ = $|S_{43}|$ = −4 dB. The results show good agreement with the corresponding four-channel results presented in Figure 9.

Table S1. Measured −3 dB bandwidth (BW_{-3dB}) in the loaded and unloaded conditions, and the corresponding BW_{-3dB} ratio (loaded/unloaded), for each channel of the two-, four-, and six-channel TMD and loop-coil configurations shown in Figure 3. Values for the 2-CH TMD configuration could not be reliably determined due to insufficient $|S_{11}|$ depth at the fundamental resonance.

	**TMD**			**Loop Coil**		
Channel Number	Loaded BW_{-3dB}[MHz]	Unloaded BW_{-3dB}[MHz]	BW_{-3dB} ratio (Loaded/ Unloaded)	Loaded BW_{-3dB}[MHz]	Unloaded BW_{-3dB}[MHz]	BW_{-3dB} Ratio (Loaded/ Unloaded)
**2-CH**						
1	Not reliably determined			10.25	1	10.25
2	Not reliably determined			8.6	2.0	4.3
**4-CH**						
1	6.8	19	0.36	3.5	1.5	2.3
2	6.8	19	0.36	3.5	1.5	2.3
3	2.1	10	0.21	3.4	1.8	1.9
4	2.2	11	0.20	2.7	1.2	2.3
**6-CH**						

1	6.9	20	0.34	5.8	3.8	1.5
2	7.0	20	0.35	3.0	1.5	2.0
3	3.0	12	0.25	4.2	2.8	1.5
4	2.9	11	0.26	2.6	1.4	1.9
5	4.0	10	0.40	2.7	1.5	1.8
6	3.9	10	0.39	2.9	1.3	2.2
